# Clinical characteristics and in-hospital prognostic factors in young patients with myocardial infarction

**DOI:** 10.3389/fcvm.2026.1864896

**Published:** 2026-10-05

**Authors:** Lin Jinxuan, Liao Yongfang, Liu Tingrong, Zhou Tao

**Affiliations:** Department of Cardiology, Southern Medical University Third Hospital, Guangzhou, Guangdong, China

**Keywords:** myocardial infarction, young patients, clinical characteristics, in-hospital outcomes, risk factors

## Abstract

**Objectives:**

This study aims to analyze the clinical characteristics and short-term prognostic factors of young patients with acute myocardial infarction.

**Methods:**

This retrospective observational study included young patients (aged 18 to 45 years) who visited our center for AMI from January 1, 2014, to October 31, 2023. Baseline and outcome data were collected through the medical records system, with the primary endpoint being in-hospital major adverse cardiovascular events (MACE). Patients were divided into MACE and non-MACE groups based on the occurrence of in-hospital MACE. Coronary angiography was performed in 154/158 patients, all achieving post-procedural TIMI 3 flow. Multivariable logistic regression using Firth penalized likelihood with *a priori*-selected variables (LVEF, serum uric acid, age) was used to identify independent predictors of MACE.

**Results:**

Of 166 young patients initially screened, 158 were included in the final analysis after excluding 8 due to incomplete data. Among these, 27 (17.1%) experienced in-hospital MACE. Patients in the MACE group had a lower prevalence of diabetes (0.00% vs. 19.08%, *P* = 0.008), lower admission blood pressure and worse left ventricular dysfunction [lower left ventricular ejection fraction (LVEF), larger left ventricular end diastolic diameter (LVEDD)]. Single-vessel disease was the most common (38.96%), with 83.77% of patients having involvement of the left anterior descending artery. The proportion of left main disease was higher in the MACE group (15.38% vs. 3.91%, *P* = 0.045). In the Firth model, lower LVEF was the sole independent predictor of MACE (OR 0.859, 95% CI 0.794–0.917, *P* < 0.001), while serum uric acid and age were not significant.

**Conclusion:**

The clinical characteristics and coronary artery lesion features of young AMI patients are unique. Reduced LVEF is the sole independent predictor of in-hospital MACE in our cohort, underscoring the value of ventricular function assessment for short-term risk stratification.

## 1 Introduction

Acute myocardial infarction (AMI) is one of the leading causes of death in humans, with an abrupt onset and a high rate of disability and mortality. In recent years, the incidence of AMI among young patients has been increasing annually (1), a trend that has drawn widespread attention from both clinical and public health communities. According to statistics, the incidence of AMI among individuals aged 20 to 29 is 2.1 per 100,000 person-years, while for those aged 30 to 39 it is 16.9 per 100,000 person-years, and for those aged 40 to 49 it is 97.6 per 100,000 person-years (2). Studies have reported that 4% to 10% of MIs occur in individuals under the age of 45 (1, 2). In China, the crude incidence rate of AMI among residents aged 18 and older is 87.6 per 100,000, with an age-standardized rate of 93.9 per 100,000 (3).

The clinical characteristics, coronary artery lesion features, and prognosis of middle-aged and young patients with MI are of great significance for guiding clinical diagnosis and treatment. However, previous studies on prognostic factors affecting middle-aged and young patients with MI have been relatively scarce, and the conclusions remain inconsistent. This study analyzes the clinical characteristics and short-term prognostic factors of MI in young patients, aiming to provide a theoretical basis for precise diagnosis and treatment in this population.

## 2 Materials and methods

### 2.1 Study population

This study is a retrospective observational study that consecutively enrolled young patients (aged 18 to 45 years) who presented to our center with AMI from January 1, 2014, to October 31, 2023.

#### 2.1.1 Inclusion criteria

Aged 18 to 45 years; patients diagnosed with AMI according to the 4th Global Consensus on the Uniform Definition of Acute Myocardial Infarction (2018) (4); complete baseline and outcome data available.

#### 2.1.2 Exclusion criteria

Severe hepatic or renal dysfunction, prior coronary revascularization, malignancy, and severe infection.

### 2.2 Data collection and definitions

Baseline characteristics, encompassing demographics, clinical profiles, and coronary angiography findings, along with in-hospital major adverse cardiovascular events (MACE), will be extracted from the electronic health record system. Baseline characteristics included demographic variables (age, gender), lifestyle factors (smoking status, alcohol consumption), comorbidities (hypertension, diabetes, hyperlipidemia, metabolic syndrome), hemodynamic parameters (systolic and diastolic blood pressure), laboratory biomarkers (serum uric acid, lipid profile [total cholesterol, triglycerides, low-density lipoprotein cholesterol], glycated hemoglobin, N-terminal pro-brain natriuretic peptide, renal function indices [serum creatinine, estimated glomerular filtration rate]), medication history (uric acid-lowering agents, diuretics), and coronary angiographic findings. The primary outcome was in-hospital MACE. Patients were stratified into MACE and non-MACE groups according to the occurrence of these events during hospitalization.

Hyperlipidemia was defined according to the *Chinese Guidelines for Lipid Management (2023)* as elevated serum cholesterol and/or triglyceride levels, specifically with thresholds of total cholesterol ≥5.2 mmol/L and/or triglycerides ≥1.7 mmol/L (5). Metabolic syndrome was defined according to the *Expert Consensus on Health Education for the Prevention and Control of Metabolic Syndrome in Adults* as the presence of ≥3 of the following 5 components: (1) abdominal obesity (waist circumference ≥90 cm for men and ≥85 cm for women); (2) elevated glucose (fasting plasma glucose ≥6.1 mmol/L, 2-hour postprandial glucose ≥7.8 mmol/L, or current treatment for diabetes); (3) elevated blood pressure (≥130/85 mmHg or current antihypertensive treatment); (4) elevated fasting triglycerides (≥1.7 mmol/L); and (5) reduced fasting high-density lipoprotein cholesterol (<1.04 mmol/L) (6). MI was classified according to the *Fourth Universal Definition of Myocardial Infarction (2018)* into five types: Type 1, spontaneous MI related to atherosclerotic plaque rupture and thrombosis; Type 2, MI secondary to ischemic imbalance unrelated to coronary thrombosis; Type 3, MI resulting in sudden cardiac death; Type 4, PCI-related MI (including stent thrombosis or restenosis); and Type 5, CABG-related MI (4). Major in-hospital cardiovascular adverse events were defined as non-fatal stroke, malignant arrhythmias, cardiac arrest, heart failure, or cardiovascular death occurring during hospitalization, excluding events that occurred before admission or after discharge.

### 2.3 Statistical analysis

Data normality was assessed using the Kolmogorov–Smirnov test. Continuous variables are presented as mean ± standard deviation or median (interquartile range, IQR), as appropriate; categorical variables are reported as frequencies and percentages. For univariate analysis, group comparisons were performed using the t-test, Mann–Whitney U test, or *χ*^2^/Fisher's exact test, according to data distribution and variable type.

Patients with Type 3 myocardial infarction (sudden cardiac death) were excluded from prognostic analyses, as death constituted an inclusion criterion rather than an outcome. Given the limited number of MACE events, multivariable logistic regression was performed using Firth penalized likelihood to reduce small-sample bias. Variables were selected *a priori* based on established prognostic relevance in young patients with myocardial infarction and included left ventricular ejection fraction (LVEF), serum uric acid, and age. Univariable logistic regression was used solely for descriptive purposes and not for variable selection. Results are expressed as odds ratios (ORs) with 95% confidence intervals (CIs). A two-sided *p* value <0.05 was considered statistically significant.

## 3 Results

### 3.1 Baseline patient characteristics

Of 166 young patients initially screened for eligibility, 158 were included in the final analysis after excluding 8 patients due to incomplete data. The median hospital stay was 7 days (IQR: 5.00–8.25). Regarding clinical outcomes, three patients died, one was transferred due to coronary-involved aortic dissection after experiencing third-degree atrioventricular block and recurrent ventricular fibrillation, and the remaining 154 were discharged after improvement.

Baseline characteristics are presented in Table 1. The study cohort comprised 158 patients, including 152 males (96.2%) and 6 females (3.8%). Notably, 2 of the 6 female patients (33.3%) experienced in-hospital MACE. A total of 27 MACEs occurred: 14 malignant arrhythmias (8.86%), 6 cardiac arrests (3.80%), 3 heart failure cases (1.90%), 3 cardiovascular deaths (1.90%), and 1 non-fatal stroke (0.63%). The most prevalent risk factors were hyperlipidemia (56.96%) and smoking (57.59%). Coronary angiography and percutaneous coronary intervention were performed in 154/158 patients (97.47%), with a 100% procedural success rate (TIMI flow grade 3). Four patients (2.5%) did not undergo revascularization. Standard secondary prevention was administered to all patients.

**Table 1:** Clinical characteristics.

Variable	All (*n* = 158)	MACE(*n* = 27)	Non-MACE(*n* = 131)	*P* value
Age (years)	39.71 (34.20，42.15)	40.51 (37.40，42.57)	39.28 (34.31，42.44)	0.303
Gender, *n* (%)				
Male, *n* (%)	152 (96.20)	25 (92.59)	127 (96.95)	0.272
Female, *n* (%)	6 (3.80)	2 (7.41)	4 (3.05)	
Smoking status, *n* (%)	91 (57.59)	13 (48.15)	78 (59.54)	0.292
Alcohol consumption, *n* (%)	54 (34.18)	9 (33.33)	45 (34.35)	1.000
Comorbidities				
Hypertension, *n* (%)	65 (41.14)	8 (29.63)	57 (43.51)	0.204
Diabetes, *n* (%)	25 (15.82)	0 (0.00)	25 (19.08)	**0**.**008**
Hyperlipidemia, (%)	90 (56.96)	13 (48.15)	77 (58.78)	0.394
Chronic kidney disease, *n* (%)	4 (2.53)	1 (3.70)	3 (2.29)	0.531
Metabolic syndrome, *n* (%)	14 (8.86)	2 (7.41)	12 (9.16)	1.000
Gout, *n* (%)	8 (5.06)	2 (7.41)	6 (4.58)	0.625
BMI (kg/m^2^)	26.65 ± 3.86	27.34 ± 3.91	25.54 ± 3.67	0.263
Admission systolic blood pressure (mmHg)	135.70 ± 23.41	117.76 ± 27.37	134.24 ± 21.10	**0**.**001**
Admission diastolic blood pressure (mmHg)	83.43 ± 18.38	72.52 ± 22.66	83.78 ± 16.85	**0**.**004**
EGFR (mL/min)	108.79 (87.60，117.73)	93.61 (68.92，110.97)	108.79 (94.69，118.21)	**0**.**011**
Serum uric acid (umol/L)	446.92 ± 112.73	459.68 ± 160.24	409.97 ± 107.61	0.206
Total cholesterol (mmol/L)	5.19 ± 1.22	5.01 ± 1.39	5.21 ± 1.33	0.507
Triglycerides (mmol/L)	1.38 (0.89，2.82)	1.08 (0.81，1.82)	1.66 (1.05，2.94)	**0**.**015**
High-density lipoprotein cholesterol (mmol/L)	1.08 (0.95，1.22)	1.16 (0.98，1.41)	1.06 (0.94，1.23)	**0**.**039**
Low-density lipoprotein cholesterol (mmol/L)	3.29 ± 1.02	3.25 ± 1.29	3.27 ± 1.07	0.936
Glycated hemoglobin (%)	5.50 (5.23，5.78)	5.55 (4.95，5.85)	5.60 (5.30，6.00)	0.182
NT-ProBNP (pg/mL)	180.50 (68.69，423.45)	365.60 (128.00，1584.50)	215.30 (76.12，451.60)	0.121
LVEDD (mm)	46.14 ± 4.20	49.08 ± 6.88	45.67 ± 4.70	**0**.**028**
LVEF (%)	60.00 (53.03，61.35)	47.45 (39.00，60.48)	60.00 (55.00，62.00)	**0**.**000**
Uric acid-lowering agents, *n* (%)	14 (8.86)	1 (3.70)	13 (9.92)	0.468
Diuretics, *n* (%)	24 (15.19)	8 (29.63)	16 (12.21)	**0**.**032**
MI types				
Type 1, *n* (%)	150 (94.94)	24 (88.89)	126 (96.18)	**0**.**007**
Type 2, *n* (%)	6 (3.80)	1 (3.70)	5 (3.82)	
Type 3, *n* (%)	2 (1.26)	2 (7.41)	0 (0.00)	
MI classification^a^				
STEMI, *n* (%)	115 (73.72)	21 (84.00)	94 (71.76)	**0**.**013**
NSTEMI, *n* (%)	41 (26.28)	4 (16.00)	37 (28.24)	
CAG + PCI, *n* (%)	154 (97.47)	24 (88.89)	130 (99.24)	0.124

MACE, major adverse cardiovascular events. EGFR, estimated glomerular filtration rate. NT-ProBNP, N-terminal pro-brain natriuretic peptide. LVEDD, left ventricular end-diastolic diameter. LVEF, left ventricular ejection fraction. STEMI, ST-Elevation Myocardial Infarction. NSTEMI, Non-ST-Elevation Myocardial Infarction, CAG, coronary angiography. PCI, percutaneous coronary intervention.

Bold values indicate *P* < 0.05 (statistically significant).

a 2 cases involving sudden cardiac death could not be classified.

Compared to the non-MACE group, patients with MACE presented with significantly lower admission blood pressure (Systolic: 117.76 ± 27.37 vs. 134.24 ± 21.10 mmHg, *P* = 0.001; Diastolic: 72.52 ± 22.66 vs. 83.78 ± 16.85 mmHg, *P* = 0.004) and eGFR [93.61 (68.92，110.97) vs. 108.79 (94.69，118.21) mL/min, *P* = 0.011]. Echocardiography revealed that the MACE group had a larger LVEDD (49.08 ± 6.88 vs. 45.67 ± 4.7 mm, *P* = 0.028) and significantly lower LVEF [47.45 (39.00，60.48) vs. 60.00 (55.00，62.00) %, *P* < 0.001), indicating worse baseline cardiac function. Interestingly, the MACE group had a lower prevalence of diabetes (0.0% vs. 19.08%, *P* = 0.008) and a higher proportion of Type 3 MI (7.41% vs. 0.0%, *P* = 0.007) and STEMI (16.00% vs. 28.24%, *P* = 0.013). Type 2 MI occurred in 6 patients (3.80%), with coronary spasm from hyperthyroidism causing 4 cases.

### 3.2 Characteristics of coronary artery lesions

Coronary angiography was performed in 150 of the 154 patients; 4 (1 MACE, 3 non-MACE) declined. As shown in Figure 1, single-vessel disease was the most common finding (60 cases, 38.96%), followed by multi-vessel disease (49 cases, 31.81%). The left anterior descending artery was involved in 129 patients (83.77%). The MACE group had a significantly higher proportion of left main lesions than the non-MACE group [4 (15.38%) vs. 5 (3.91%), *P* = 0.045]. However, no significant differences were observed between the groups in the involvement of the left anterior descending artery [21 (80.77%) vs. 108 (84.38%), *P* = 1.000], circumflex artery [9 (31.62%) vs. 55 (42.97%), *P* = 0.659], or right coronary artery [11 (42.31%) vs. 74 (57.81%), *p* = 0.494], nor in the number of diseased vessels (*P* = 0.673; Figure 1).

**Figure 1:**
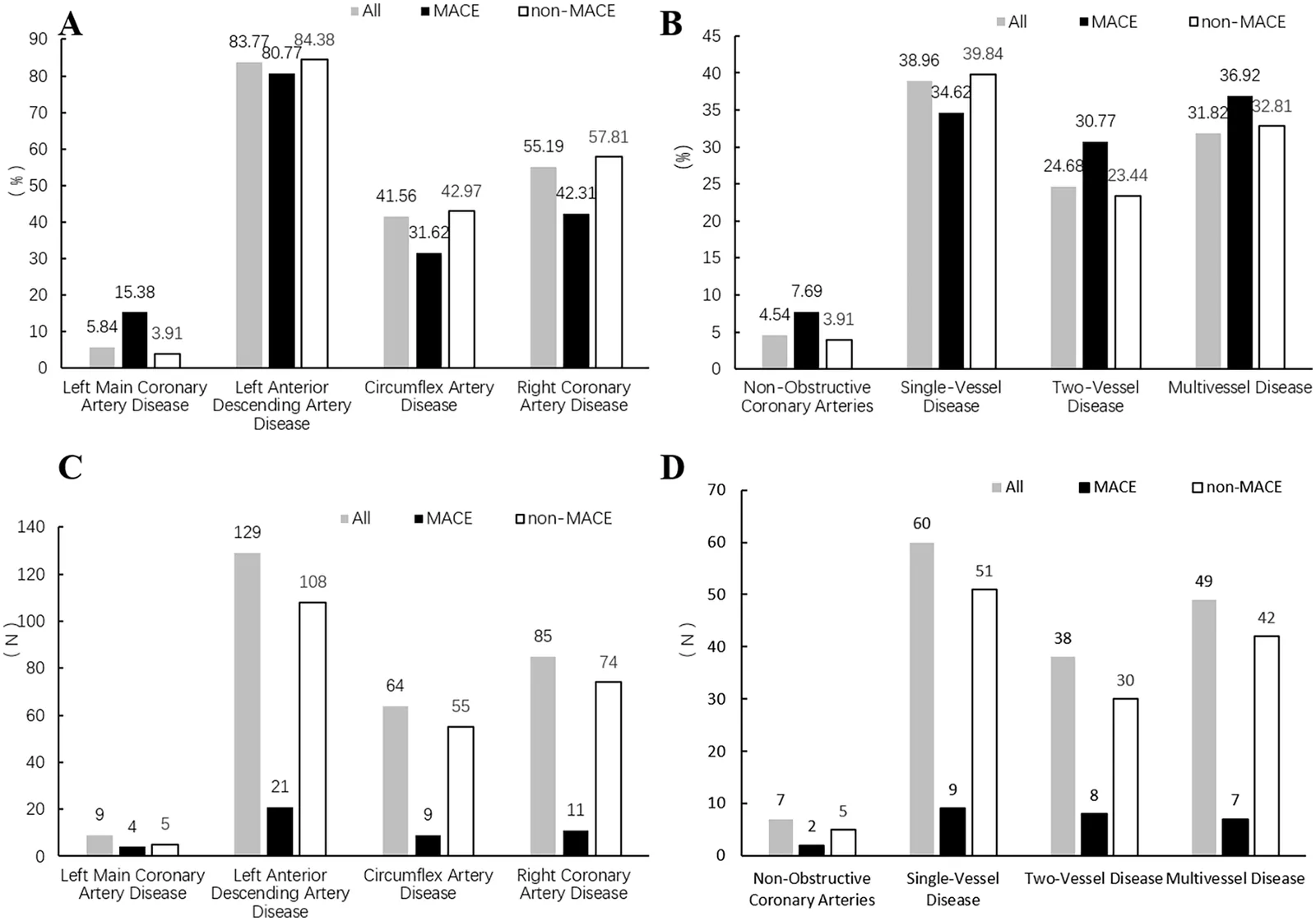
Characteristics of coronary artery lesions. Panels **(A,C)** show the proportion and number of lesion locations; Panels **(B,D)** show the number of diseased vessels. MACE, major adverse cardiovascular events. Four patients (1 MACE, 3 non-MACE) were excluded for refusing angiography.

### 3.3 Short-term prognostic factors

Two patients (7.41% of the MACE group) were diagnosed with type 3 myocardial infarction. These cases were included in baseline characteristics but excluded from regression models. In the multivariable Firth penalized logistic regression model, lower LVEF remained an independent predictor of MACE (OR 0.859, 95% CI 0.794–0.917, *P* < 0.001), whereas serum uric acid (OR 1.002, 95% CI 0.998–1.007, *P* = 0.336) and age (OR 1.098, 95% CI 0.966–1.278, *P* = 0.163) were not significantly associated with MACE (Figure 2). All other variables listed in Table 1 were analyzed by univariable logistic regression only and are reported descriptively (Supplementary Table S1).

**Figure 2:**
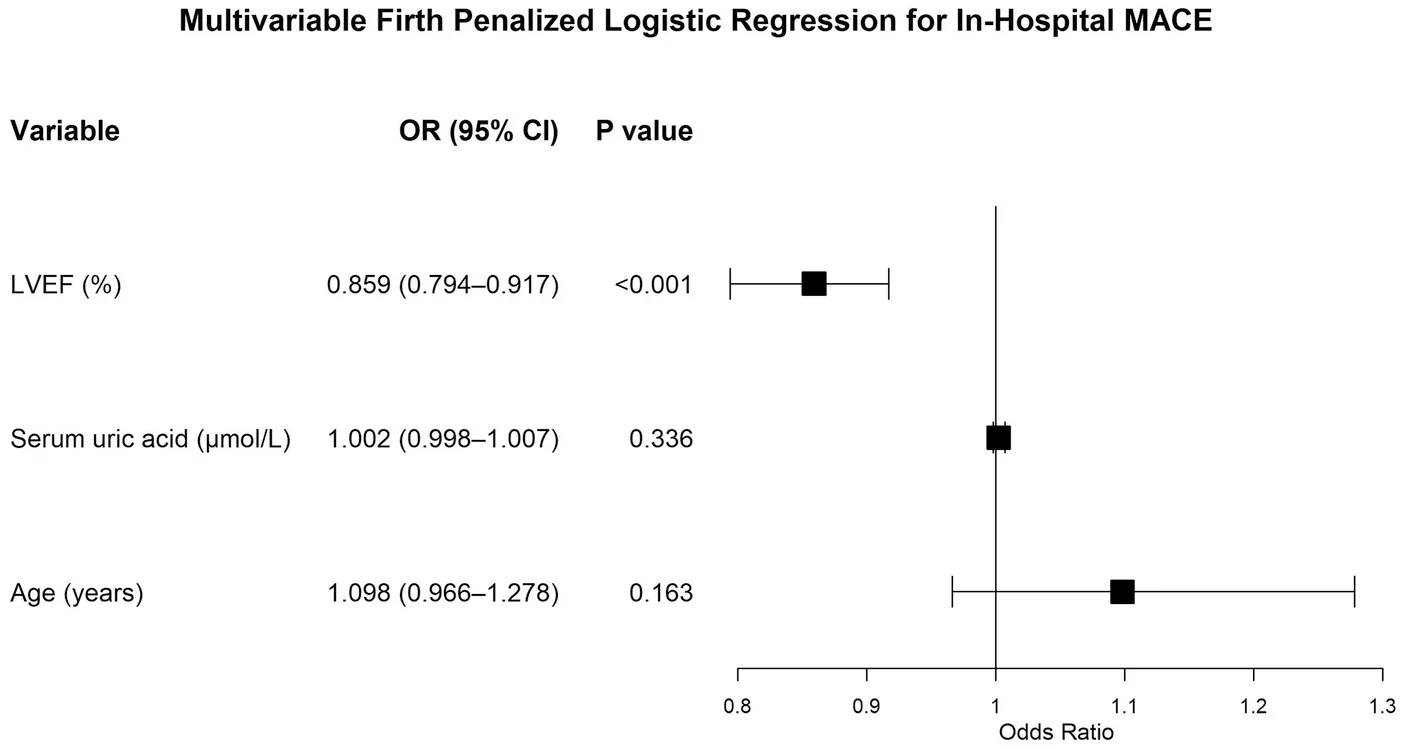
Analysis of risk factors for MACE in young patients with MI. OR, odds ratios. CIs, confidence intervals.

## 4 Discussion

Myocardial infarction (MI) in young adults remains a critical public health concern due to its substantial clinical and socioeconomic burden. Distinct from the elderly population, this demographic exhibits unique clinical phenotypes and prognostic trajectories, likely driven by specific risk profiles, lifestyle factors, and pathophysiological mechanisms (7, 8). Given the escalating incidence of MI in this cohort, elucidating its clinical characteristics and determinants of prognosis is imperative for optimizing early intervention strategies and improving long-term outcomes.

In our cohort, males accounted for 96.2% of the study population, with a median age of onset between 30 and 40 years. This high proportion of male patients is consistent with the well-established epidemiology of young MI, where prior literature has reported male prevalence ranging from 81% to 92.1% (2, 9, 10). Contrary to typical atherosclerotic risk patterns, diabetes was absent in the MACE group (0.00% vs. 19.08%, *P* = 0.008), which presented with lower admission blood pressure and worse baseline cardiac function (lower LVEF, larger LVEDD). This seemingly paradoxical finding likely reflects that our in-hospital MACE—dominated by malignant arrhythmias and cardiac arrest—was driven by acute hemodynamic collapse rather than chronic atherosclerotic burden.​ The higher prevalence of left main coronary artery disease in the MACE group (15.4% vs. 3.9%, *P* = 0.045) further underscores the severity of acute insult. Nevertheless, this observation should be interpreted cautiously due to the small absolute number of diabetic patients in the MACE subgroup (*n* = 0).

In the present study, reduced LVEF was the only independent predictor of in-hospital MACE (OR 0.859, 95% CI 0.794–0.917, *P* < 0.001) among young patients with acute myocardial infarction. This finding aligns with large-scale registry evidence (11–14). Reitan et al. (11), analyzing over 330,000 MI patients in the nationwide SWEDEHEART registry, demonstrated that reduced LVEF was associated with the greatest loss of life expectancy, particularly in younger patients. Similarly, Ge et al. (12) developed and validated the BACEF risk score—comprising serum albumin, age, creatinine, and LVEF—confirming LVEF as one of the four strongest predictors of 4-year mortality. Notably, our cohort achieved a 100% procedural success rate (TIMI 3 flow) in PCI-treated patients, suggesting that the prognostic impact of LVEF in our study was driven by the severity of initial myocardial injury rather than suboptimal reperfusion.

Consistent with prior reports (15–17), single-vessel disease predominated in our cohort (38.96%), with the left anterior descending artery being the most frequently involved vessel (83.77%). This distribution is consistent with a prospective observational study of young STEMI patients (≤45 years) in which single-vessel disease was observed in 50% and LAD was the culprit artery in 67.3% of cases (15). Similarly, Nozhov et al. (16) reported single-vessel disease in 41.9% of young MI patients (<44 years) and non-obstructive lesions in 4.3%, underscoring the heterogeneity of coronary atherosclerosis in this demographic. Ewa M et al. (17) similarly reported that single-vessel disease accounts for 61.9% of MIs in young patients, with the left anterior descending artery being the most frequently affected site (61.6%). The relatively low proportion of multi-vessel disease in our cohort (31.81%) aligns with the prevailing single-vessel phenotype observed in young MI populations (15–18). Notably, type 2 MI accounted for 3.8% (6/158) of cases, a prevalence comparable to prospective European multicenter data (19). Consistently with Rallidis et al. (20), who reported a notable proportion of young STEMI patients with normal or near-normal coronary arteries and emphasized non-atherosclerotic etiologies, our results underscore the heterogeneity of coronary lesion profiles in this demographic.

## 5 Conclusion

In this retrospective cohort of young patients with AMI, we identified a high prevalence of traditional risk factors and a distinct coronary phenotype characterized by single-vessel LAD involvement. Crucially, reduced LVEF emerged as the sole independent predictor of in-hospital MACE. Crucially, reduced LVEF was identified as an independent risk factor for poor prognosis, underscoring the need for risk stratification based on ventricular function in this demographic.

## 6 Limitations

This study has several limitations inherent to its single-center retrospective design, which may introduce selection bias and limit generalizability. Notably, the low proportion of female patients (3.8%) reflects the known epidemiology of MI in young populations; thus, we restricted our analysis to descriptive statistics and did not perform independent hypothesis testing for gender differences. Given the limited number of MACE events (*n* = 27), a full multivariable model would violate the 10-events-per-variable rule. We therefore employed a parsimonious, *a priori* three-variable Firth model. Accordingly, our findings should be considered exploratory and warrant validation in larger, multicenter studies.

## Data Availability

The original contributions presented in the study are included in the article/Supplementary Material, further inquiries can be directed to the corresponding author/s.
