# Supplementary material for: Clinical characteristics and in-hospital prognostic factors in young patients with myocardial infarction
Source: Front Cardiovasc Med. 2026 Oct 5;13:1864896. doi: 10.3389/fcvm.2026.1864896 (PMC13677364; doi:10.3389/fcvm.2026.1864896)
Supplement: Supplementary file 1 [file Table1.docx]

**Table S1. Univariate logistic regression analysis of factors associated with MACE**

| **Variable** | **OR (95% CI)** | **P value** |
| --- | --- | --- |
| *Age, years* | 1.050 (0.963–1.150) | 0.324 |
| *Gender* | 0.362 (0.067–2.720) | 0.257 |
| *Smoking* | 0.736 (0.310–1.760) | 0.484 |
| *Alcohol use* | 1.070 (0.425–2.580) | 0.874 |
| *Hypertension* | 0.505 (0.186–1.240) | 0.154 |
| *Diabetes mellitus* | **NE** | 0.990 |
| *Hyperlipidemia* | 0.647 (0.271–1.530) | 0.321 |
| *Chronic kidney disease* | 1.780 (0.086–14.600) | 0.625 |
| *Metabolic syndrome* | 0.862 (0.129–3.440) | 0.853 |
| *Gout* | 0.868 (0.045–5.400) | 0.898 |
| *BMI, kg/m²* | 1.150 (0.908–1.490) | 0.262 |
| *SBP, mmHg* | 0.961 (0.936–0.983) | 0.002 |
| *DBP, mmHg* | 0.957 (0.924–0.985) | 0.006 |
| *SUA, μmol/L* | 1.000 (0.999–1.010) | 0.089 |
| *Total cholesterol, mmol/L* | 0.890 (0.624–1.240) | 0.504 |
| *Triglycerides, mmol/L* | 0.656 (0.395–0.947) | 0.060 |
| *HDL-C, mmol/L* | 0.984 (NA–1.030) | 0.759 |
| *LDL-C, mmol/L* | 0.983 (0.648–1.470) | 0.935 |
| *HbA1c, %* | 0.562 (0.267–0.943) | 0.079 |
| *NT-proBNP, pg/mL* | 1.000 (1.000–1.000) | 0.247 |
| *LVEDD, mm* | 1.110 (1.030–1.210) | 0.008 |
| *LVEF, %* | 0.858 (0.801–0.910) | <0.001 |
| *Diuretic use* | 3.380 (1.220–9.000) | 0.016 |
| *Urate-lowering therapy* | 0.378 (0.020–2.040) | 0.360 |
| *MI type (2 vs 1)* | 1.050 (0.054–6.900) | 0.965 |
| *STEMI vs NSTEMI* | 2.070 (0.727–7.440) | 0.210 |

**P<0.05, **P<0.01, ***P<0.001. NE = not estimable (complete separation).*

*Firth penalized logistic regression was used throughout (logistf package, R).*

*Patients with type 3 MI were excluded from all regression analyses.*
